# The Role of Direct-Acting Antivirals in Enhancing Quality of Life Among Individuals with Chronic Hepatitis C

**DOI:** 10.3390/healthcare13080878

**Published:** 2025-04-11

**Authors:** Mihail Cristian Pirlog, Claudia Monica Danilescu, Dragos Ovidiu Alexandru, Costin Teodor Streba, Ion Rogoveanu

**Affiliations:** 1 Medical Sociology Department, Faculty of Medicine, University of Medicine and Pharmacy of Craiova, 200349 Craiova, Romania; mihail.pirlog@umfcv.ro; 2 Faculty of Nursing, University of Medicine and Pharmacy Craiova, 200349 Craiova, Romania; 3 Biostatistics Department, Faculty of Medicine, University of Medicine and Pharmacy of Craiova, 200349 Craiova, Romania; dragos.alexandru@umfcv.ro; 4 Department of Scientific Research Methodology, Faculty of Medicine, University of Medicine and Pharmacy of Craiova, 200349 Craiova, Romania; costin.streba@umfcv.ro; 5 Department of Gastroenterology, Faculty of Medicine, University of Medicine and Pharmacy of Craiova, 200349 Craiova, Romania; ion.rogoveanu@umfcv.ro

**Keywords:** hepatitis C, chronic, direct-acting antivirals, health-related quality of life, sustained virologic response, psychological distress, single-center study

## Abstract

**Background:** Chronic hepatitis C virus (HCV) infection significantly impairs health-related quality of life (HRQoL) and poses a substantial global health concern. Direct-acting antiviral (DAA) therapies have revolutionized HCV treatment, but their impact on HRQoL, particularly considering clinical and psychological factors, requires further investigation. This study aimed to evaluate the influence of DAA therapy on HRQoL in Romanian patients with chronic HCV infection, analyzing the effects of treatment on HRQoL and the role of associated factors. **Methods:** A prospective, single-center study was conducted on 90 HCV-infected patients treated with a 12-week DAA regimen (Ombitasvir/Paritaprevir/Ritonavir/Dasabuvir). HRQoL was assessed at baseline (BSL), end of treatment (EOT), and 12 weeks post-treatment (SVR) using the WHOQOL BREF questionnaire. Clinical data, including fibrosis degree, prior PegIFN treatment, and psychological assessments (HADS, PSS), were collected. Statistical analyses examined HRQoL trends and associations with clinical and psychological parameters. **Results:** Significant improvements in HRQoL were observed across all domains over time (*p* < 0.0001). Gender and residence did not significantly influence HRQoL changes. Fibrosis severity and prior PegIFN treatment had no significant impact on HRQoL progression. However, comorbidities such as anemia and chronic kidney disease moderated improvements in specific HRQoL domains. Anxiety also affected HRQoL, while perceived stress and depression did not show significant effects. **Conclusions:** DAA therapy significantly enhances HRQoL in HCV-infected patients. While clinical and treatment-related factors had limited influence, comorbidities and anxiety played a moderating role. These findings underscore the importance of personalized care and integrated mental health assessments in HCV management.

## 1. Introduction

Chronic hepatitis C virus (HCV) infection constitutes a major global health concern, with a global prevalence of HCV varying between 1 and 3%, and approximately 170 million individuals worldwide affected [1,2,3]. HCV infection is a leading etiological factor in the development of liver cirrhosis, hepatic decompensation, and hepatocellular carcinoma, contributing significantly to public health challenges and socioeconomic burdens [4,5,6].

Beyond hepatic involvement, HCV is associated with a wide spectrum of extrahepatic manifestations, including type 2 diabetes mellitus (T2DM), hyperlipidemia, cryoglobulinemia, lymphoma, atherosclerosis, ischemic heart disease, glomerulonephritis, and various autoimmune disorders [7,8,9,10]. Additionally, HCV is linked to a higher prevalence of mental health conditions such as mood disorders [9,11,12,13], cognitive impairment [14,15], and psychological distress [16,17,18]. This broad range of comorbid conditions is also associated with symptoms such as severe fatigue, joint pain, headaches, irritability, itching, sleep disturbances, poor appetite, and anorexia [19,20,21]. These symptoms often lead to functional disability and a severely impaired quality of life, resulting in significant social and economic consequences, including loss of work productivity, reduced daily functioning, and diminished well-being [22,23].

The assessment of patients’ health-related quality of life (HRQoL) has become an increasingly important focus in public health, medical research, and clinical management [24,25]. Today, measuring HRQoL as a subjective, multidimensional concept is considered one of the most effective methods for evaluating the impact of chronic diseases, surgical procedures, or therapeutic interventions [26].

The wide range of symptoms associated with liver infection, both directly from the disease itself and from extrahepatic manifestations [27,28], leads to significantly lower HRQoL among patients with HCV infection compared to both the general population and individuals with chronic hepatitis B [29,30].

In this context, addressing the impairment in HRQoL caused by HCV infection has become a critical target of HCV therapeutic strategies. However, accurately assessing HRQoL in patients with this condition presents a significant challenge, particularly due to its frequently asymptomatic nature [31].

Recent advancements in HCV treatment have marked a significant transition from interferon (IFN)-based regimens to direct-acting antiviral (DAA) therapies, which demonstrate superior efficacy [32]. These therapies effectively target both hepatic and extrahepatic complications, making IFN-free DAAs the principal modern HCV treatment. They achieve remarkably high sustained virologic response (SVR) rates, with 96% of patients reaching SVR at 12 weeks post-therapy [33]. In addition, DAAs are characterized by shorter treatment durations, fewer side effects [34], a lower risk of mortality and hepatocellular carcinoma (HCC) [32], excellent tolerability [32], and significant improvements in managing extrahepatic manifestations [35,36,37].

The effects of the new DAA therapies on HRQoL have not been extensively studied so far [38], despite substantial evidence of their efficacy in curing the disease. To address this, previous studies have utilized various instruments, both general and specific, such as the Short-Form Health Survey (SF-36) [39], the Hepatitis Quality of Life Questionnaire (HQLQ) [40], the Chronic Liver Disease Questionnaire (CLDQ) [41], the Liver Disease Quality of Life Instrument [42], and the Chronic Liver Disease Quality of Life (CLDQOL) Questionnaire [43]. While evidence has shown that IFN-based regimens negatively impact quality of life [22,44,45], the positive effects of DAAs in this domain were demonstrated as early as the registration trials, where improvements in patients’ HRQoL were measured [46]. Further research has highlighted substantial improvements in HRQoL during the first month of DAA therapy [47,48], but especially following the achievement of SVR [20,49,50,51], accompanied by a notable positive impact on economic productivity [52,53,54]. However, there were also studies that concluded that there were no significant changes in the quality of life after the DAA treatment, while findings from cumulative studies have been inconclusive [55,56,57].

In the context of the complex burden that HCV infection imposes on both affected individuals and society, we considered it very important to evaluate how a 12-week interferon-free direct-acting antiviral (DAA) regimen impacts health-related quality of life (HRQoL) in Romanian patients with chronic hepatitis C (HCV), and what are the associated clinical and psychological factors influencing these changes. Thus, we hypothesized that DAA therapy leads to significant improvements in HRQoL, particularly in physical and psychological well-being, but a series of factors may moderate the extent of these improvements during and after a 12-week DAA treatment period, which led us to explore the relationship between subjective perceptions of life’s quality and the clinical outcomes of this new therapeutic approach in a real-life setting, as well as the influence of previously identified psychological factors [13,18] on the progression of HRQoL. To our knowledge, this study is the only one conducted on a population of Romanian individuals with HCV infection undergoing DAA treatment, focusing on assessing the treatment’s benefits on the psychological and social aspects involved in this process.

## 2. Materials and Methods

We have carried out a prospective, single-center study on a sample consisting of 90 individuals diagnosed with HCV infection (genotype GT-1b subtype), presenting with either compensated hepatic cirrhosis (Child–Pugh class A or B) or hepatitis, between 1 August 2017 and 31 December 2018 at the Gastroenterology Clinic of the Emergency Hospital in Craiova, Dolj County, Romania [13,18]. All participants underwent a 12-week treatment with direct-acting antivirals (Ombitasvir/Paritaprevir/Ritonavir/Dasabuvir), administered in accordance with the Romanian National Protocol for HCV-infected patients [58], and the guidelines of the Romanian Society of Gastroenterology and Hepatology [59,60]. The patients were recruited from those enrolled in the Romanian national program for interferon-free HCV therapy, age > 18 years, and treatment-naïve or with prior interferon-based therapy. The inclusion criteria mentioned that they had no neurological or psychiatric comorbidities at the moment of recruitment, and had not been on psychiatric medication for the past 12 months. Personal medical history data were collected during the initial evaluation visit required for eligibility in the Romanian National Program for HCV IFN-free therapy. At the study’s baseline, the gastroenterologist reviewed each patient’s medical history, verified personal records, and conducted a structured clinical interview to assess their psychiatric status, ensuring compliance with the study’s inclusion criteria.

The biological and psychological status of the patients was evaluated at three key time points during the study:

- Baseline (BSL), prior to initiating DAA treatment;
- End of treatment (EOT), at the conclusion of the 12-week DAA treatment period;
- Follow-up (SVR), twelve weeks after completing treatment, to assess the sustained virologic response.

Subjects underwent a clinical evaluation and detailed medical history, including previous interferon-based treatment and comorbidities. Demographic data (gender, age, residence), clinical measurements (height, weight, BMI), and biological parameters (FibroTest^®^, FibroScan^®^ (FibroScan 502 Touch, Echosense, Paris, France), HCV-RNA, Child scores, and blood tests) were recorded. Fibrosis levels were classified according to international guidelines (F0–F4), and blood samples were analyzed for biomarkers such as hemoglobin, ALT, AST, bilirubin, GGT, albumin, alpha-fetoprotein, creatinine, and INR.

Depression and anxiety were assessed using the Hospital Anxiety and Depression Scale (HADS), a self-reported screening tool comprising 14 multiple-choice questions: seven for the depression subscale (HADS-D) and seven for the anxiety subscale (HADS-A), scored on a four-point Likert scale (0–3), with higher scores indicating greater severity (0–7 = non-case, 8–10 = possible case (borderline), and 11–21 = probable case) [61,62,63]. The version validated for the Romanian population was used [64].

Psychological distress was measured using the Perceived Stress Scale (PSS), the original 14-item version, with 7 positive and 7 negative items, rated on a 5-point Likert scale (0–4). The cut-off values were as follows: 0–13 (low stress), 14–26 (moderate stress), and 27–40 (high perceived stress) [65]. The Romanian version demonstrated good internal consistency (Cronbach’s alpha > 0.70) and reliability (Pearson’s, Spearman’s, or intraclass correlation > 0.70) [66].

The subjects’ quality of life was assessed by the WHOQOL BREF, a 26-item instrument consisting of four domains: physical health (7 items), psychological health (6 items), social relationships (3 items), and environmental health (8 items); it also contains QOL and general health items. Each individual item of the WHOQOL BREF is scored from 1 to 5 on a response scale, which is stipulated as a five-point ordinal scale. The scores are then transformed linearly to a 0–100 scale, where higher scores mean a better perceived quality of life [67,68]. The WHOQOL BREF is a widely recognized instrument designed for cross-cultural comparisons of quality of life and is available in over 40 languages, including Romanian [69].

Surveys were completed by patients during visits at each assessment point, following explanations by the gastroenterologist and research assistant. The data were securely recorded in a dedicated digital database.

Data collection was consistent across all three evaluation moments (BSL, EOT, SVR).

As participants were enrolled in the Romanian National Program for HCV interferon-free therapy, no drop-outs were recorded during the study period, and assessments coincided with the national program’s schedule.

Participation was voluntary, and informed consent was obtained. The study was approved by the Ethics Committee of University of Medicine and Pharmacy of Craiova (Ethical Approval of Research Project no. 66/23 February 2017), adhering to the Helsinki Declaration.

### Statistical Analysis

The study database included all items recorded during the research process and consisted of a Microsoft Excel file. The study database, stored as a Microsoft Excel file, contained all recorded data. All statistical analyses were performed by SPSS version 25 (IBM Corp., Armonk, NY, USA).

The descriptive analysis of the results was performed based on the absolute and relative frequencies (%) for the continuous variables, as well as the mean standard deviation for normally distributed data.

Group differences were analyzed using the Friedman test for repeated measures when the dependent variable was ordinal, as well as the Mann–Whitney U and Kruskal–Wallis H tests for pairwise and multi-group comparisons, respectively. Categorical data were evaluated using the Chi-square test (χ^2^) or Fisher’s exact test for small samples. Additionally, the Shapiro–Wilk test was employed to assess data normality, and Levene’s test was used to evaluate homogeneity of variance. The following *p*-value thresholds were considered acceptable: *p* < 0.05 (significant, 95% confidence interval [CI]), *p* < 0.01 (significant, 99% CI), and *p* < 0.001 (highly significant, 99.9% CI).

The study’s statistical power, determined by its sample size, was calculated using G*Power 3.1.9.7 software (Heinrich Heine University Düsseldorf, Düsseldorf, Germany). The analysis indicated a minimum required sample size of 42 participants, which increased to 49 participants after accounting for a 15% correction. This calculation was based on an effect size (Kendall’s W) of 0.2, a significance level (α) of 0.05, and a statistical power (1 − β) of 0.8, applied to Friedman’s test for three groups of repeated measures.

## 3. Results

According to the age and gender of the subjects, the study sample included 18.84% females and 23.81% males under 60 years, 60.87% females and 47.62% males between 60 and 69 years, and 20.29% females and 28.57% males between 70 and 79 years. No significant gender differences were observed across age groups (*p* = 0.553). Regarding residence, 55.07% of females and 47.62% of males lived in rural areas, and 44.93% of females and 52.38% of males lived in urban areas. The distribution of residence was not statistically significant (*p* = 0.549) (Table 1).

The clinical data included participants’ fibrosis degree and prior PegIFN treatment. Regarding fibrosis degree, the majority of subjects were classified as F4 (55.07% of females and 71.43% of males), with no significant differences observed between genders (*p* = 0.314). Additionally, 27.54% of females and 33.33% of males had a history of PegIFN treatment, with no statistically significant difference noted (*p* > 0.05) (Table 2).

The study evaluated the side effects of the current DAA therapy. At EOT, fatigue was reported by 8.70% of females and 9.52% of males, headache by 7.25% of females and 4.76% of males, insomnia by 7.25% of females and none of the males, and pruritus by 10.14% of females and 19.05% of males. No significant gender differences were observed for these side effects (*p* > 0.05). At the SVR phase, only fatigue and pruritus were reported, with no statistically significant differences between genders (*p* > 0.05) (Table 3).

We evaluated the presence of comorbidities among participants. For obesity, 76.81% of females and 66.67% of males were affected, with a total of 74.44% of participants having a certain degree of obesity (*p* > 0.05). Regarding anemia, 91.30% of females and 47.62% of males were affected (*p* > 0.05), while 91.30% of females and 42.86% of males had chronic kidney disease (CKD), with a significant gender difference observed (*p* = 0.0000012 *p* < 0.001). The other two comorbidities recorded were high blood pressure, present in 63.77% of females and 61.90% of males (*p* = 0.877), and diabetes mellitus, affecting 20.29% of females and 4.76% of males (*p* = 0.095) (Table 4).

According to the study’s methodology, we assessed perceived stress levels (PSS), as well as the presence of anxiety and depression across the three stages of treatment. At BSL, 75.81% of females and 85.71% of males reported moderate stress (*p* > 0.05). This trend was maintained at EOT, when 78.26% of females and 61.90% of males reported moderate stress (*p* = 0.109), and at SVR, when 69.57% of females and 52.38% of males reported moderate stress (*p* = 0.092).

Anxiety, evaluated through HADS-A scores, showed a decreasing trend between the first and final assessments. At BSL, 46.38% of females and 33.33% of males had anxiety (*p* = 0.338), while at SVR, this number significantly decreased (only two females and two males reported anxiety symptoms), with no significant gender differences (*p* > 0.05).

Depression, as measured by HADS-D, was present in 17.39% of females and 33.33% of males at BSL, with an improvement by the final assessment (only one male still exhibited depressive symptoms) (*p* > 0.05) (Table 5).

The participants’ quality of life (QoL) was measured using the WHOQOL BREF scale across three stages of treatment. Significant improvements were observed in all domains (Overall QoL and general health; Physical; Psychological; Social; Environment) over time (*p* < 0.0001 for all comparisons), for both sexes and for the whole study sample as well. These improvements across all domains highlight the significant positive impact of DAA treatment on the quality of life of individuals with HCV infection (Table 6).

We compared the WHOQOL BREF domains across different stages of treatment, considering gender and residence. For Overall QoL and general health, there were no significant differences between the stages for both gender and residence (*p* > 0.05). The same pattern was observed for all other instrument’s domains, where no significant differences were found between EOT vs. BSL, SVR vs. EOT, and SVR vs. INIT across either gender or residence (*p* > 0.05 for all comparisons). These findings suggest that the changes in quality of life across stages were consistent regardless of gender or residence (Table 7).

We also assessed the differences in QOL based on fibrosis degree and prior PegIFN treatment. The fibrosis degree did not significantly influence the trend of WHOQOL BREF scores between stages (*p* > 0.05). Similarly, no significant differences were found regarding the impact of prior PegIFN treatment on QOL during the study (*p* > 0.05), except for the Environment domain between EOT and SVR (*p* = 0.072), which approached statistical significance. The presence and severity of DAA therapy side effects were reflected in the Social (SVR vs. EOT, *p* = 0.048) and Environment domains (SVR vs. EOT, *p* = 0.015, and EOT vs. BSL, *p* = 0.016) assessed by the WHOQOL BREF. No other significant differences were found (*p* > 0.05). Based on these results, we conclude that neither prior treatment of HCV infection nor the severity of liver damage had a significant impact on the quality of life of our study participants. Rather, the side effects of the current therapy significantly affected the Social and Environment domains during the research period (Table 8).

Our analysis included the assessment of differences in the QOL based on the presence of identified comorbidities (obesity, anemia, chronic kidney disease, high blood pressure, and diabetes). For obesity, no significant differences were observed (*p* > 0.05), except in the Environment domain, where significant differences were found between EOT and BSL (*p* = 0.047) and SVR vs. EOT (*p* = 0.044). For anemia, as the quality of life improved during the treatment period, significant differences were noted in several domains: Physical (SVR vs. EOT, *p* = 0.017), Psychological (SVR vs. EOT, *p* = 0.018), and Environment (SVR vs. EOT, *p* = 0.003). For CKD, significant differences were found only in the Social domain (EOT vs. BSL, *p* = 0.003), with a trend toward improvement between stages. High blood pressure and diabetes did not significantly influence the trend of QOL across stages (*p* > 0.05). These results suggest that comorbidities such as anemia and chronic kidney disease had a more noticeable effect on certain aspects of quality of life during treatment compared to others, while obesity, high blood pressure, and diabetes proved to have a minimal impact (Table 9).

Finally, we evaluated the differences in WHOQOL BREF domains based on the psychological status (perceived stress, anxiety, and depression) of individuals with HCV infection undergoing DAA treatment. For perceived stress, no significant differences were found (*p* > 0.05), except in the Physical domain between SVR and BSL (*p* = 0.032). Anxiety led to significant differences in the Psychological (EOT vs. BSL, *p* = 0.002) and Environment (EOT vs. BSL, *p* = 0.021) domains, with improvements in quality of life observed. No significant differences were observed in other domains (*p* > 0.05). For depression, no significant differences were found in any of the WHOQOL BREF domains across stages (*p* > 0.05 for all comparisons). These results suggest that perceived stress and anxiety have a notable effect on certain quality of life domains during treatment, particularly in the Psychological and Environmental aspects, while depression did not show a significant impact across stages (Table 10).

## 4. Discussion

Our study demonstrated that DAA therapy significantly improved HRQoL, though gender and comorbidities moderated this effect. Prior treatments and liver damage severity did not influence HRQoL, but clinicians should address both side effects, even if DAA therapy exhibits fewer and less severe consequences than previous treatments, and the psychological impacts of therapy, as they significantly affect HRQoL.

Chronic HCV infection remains a major global health concern, as evidenced by its high prevalence [1,2] and its association with severe hepatic and extrahepatic complications [7,9]. Our research aimed to explore the relationship between subjective perceptions of quality of life and the clinical outcomes associated with the new DAAs therapeutic approach, as well as the influence of previously identified psychological factors [13,18]. Evaluating how quality of life evolves during and after DAA treatment is extremely important, given the complex burden HCV infection imposes on both affected individuals and society [6]. The introduction of DAAs as the primary therapeutic approach in HCV management has marked significant progress, with impressive SVR rates exceeding 95% [33], shorter treatment durations and a lower side-effect profile [34]. Additionally, other studies have noted substantial improvements in patient-reported outcomes following DAA treatment [34,46]. In this context, the outcomes of our study reaffirm the importance of HRQoL as a key outcome in the management of HCV infection.

According to the trend of WHOQOL BREF scores over the study period, substantial improvements were observed across all domains of HRQoL, highlighting the therapeutic benefits of DAA therapy (*p* < 0.0001). Enhancements in the Physical and Psychological domains were particularly pronounced, indicating relief from disease-related symptoms and psychological distress. These findings align with previous research emphasizing the positive impact of this treatment [20,46,48,49,51].

Our study highlighted significant gender differences and the impact of comorbid conditions in the context of liver disease. For example, CKD affected 70% of female participants with HCV infection (*p* = 0.0000012), significantly impairing the Social domain of the WHOQOL BREF (*p* = 0.003). This finding contrasts with prior studies that report a higher vulnerability of men to renal disease in the context of chronic liver infection [70,71]. Other notable comorbidities included anemia and obesity. In our study, anemia adversely affected the Physical and Psychological domains of the WHOQOL BREF (*p* = 0.017 and *p* = 0.018, respectively), aligning with previous findings that emphasize its negative impact on the perceived quality of life [72,73]. Obesity, which was present in 74.44% of participants, highlights the interplay between metabolic conditions and HCV, as supported by literature linking HCV to an increased risk of metabolic syndrome [74,75]. Despite its high prevalence, weight gain had a minimal impact on HRQoL trends, suggesting that its severity may be influenced by effective DAA therapy. These findings emphasize the importance of personalized care strategies that address gender-specific health challenges and advocate for a multidisciplinary approach to patient care [76].

Regarding the degree of fibrosis at the beginning of treatment with the new group of antiviral drugs, the extent of liver damage among our study participants did not significantly influence their subjective perception of quality of life. This finding aligns with the existing literature [77,78]. A similar trend was observed regarding the impact of prior PegIFN treatment on WHOQOL BREF scores across treatment stages, except for a near-significant difference in the Environment domain (*p* = 0.072). This contrasts with studies suggesting that interferon-based therapy may exacerbate HRQoL impairments [44,45]. This discrepancy may be attributed to the homogeneity of the study sample or the highly effective DAA treatment, which may mitigate the negative effects of disease severity or prior treatment experiences.

Despite the clear positive outcomes of DAA therapy, challenges remain regarding residual side effects. Participants in our study reported symptoms such as fatigue and pruritus even after achieving SVR, albeit at a reduced incidence, in line with data provided by other articles [79,80]. Although not statistically significant, these symptoms can affect patients’ daily lives, highlighting the need for ongoing monitoring and adjunctive treatments in order to reduce their negative effects.

In our studied population, psychological status is a critical determinant of quality of life, and a significant improvement was observed in the Psychological domain of the WHOQOL BREF. While perceived stress levels during treatment were not significant factors influencing how patients’ lives were affected, anxiety, as measured by HADS-A scores, showed a marked decrease from baseline to SVR. This improvement was associated with significant gains in the Psychological and Environmental domains of the assessment tool. These findings align with previous studies highlighting the positive impact of DAA therapy on mental health outcomes [38,81].

Depressive disorder, even if it was present among individuals diagnosed with HCV infection, did not have a significant effect on their quality of life, despite some individuals reporting depressive symptoms at SVR. Similar findings have been reported in other studies, underscoring the need for psychological interventions during and after antiviral therapy to achieve improved outcomes and enhance overall HRQoL [82,83].

The Social and Environmental domains assessed by the WHOQOL BREF also demonstrated significant improvement, highlighting the broader benefits of DAA therapy. Improvements in these domains reflect enhanced interpersonal relationships and better social functioning, which may result from reduced stigma, alleviation of disease-related restrictions, and improved financial stability and access to healthcare resources. These findings align with previous studies linking successful HCV treatment to better socioeconomic outcomes and reduced work productivity losses [52,54].

We believe our study offers valuable contributions by providing a detailed analysis of the multifaceted factors influencing HRQoL in HCV-infected patients undergoing DAA therapy within a Romanian population. Notably, we present a unique examination of gender-specific impacts of comorbidities on HRQoL. Furthermore, our study represents the first analysis of the interplay between psychological and social factors and their influence on treatment response within this patient group. While our longitudinal design and the use of validated instruments offer valuable insights into the impact of DAA therapy on HRQoL in HCV-infected patients, we acknowledge several limitations. Firstly, the absence of a control group restricts our ability to definitively attribute the observed HRQoL improvements solely to the DAA treatment. Secondly, although this research represents an initial investigation into the impact of DAAs on HRQoL within the Romanian context, the relatively small sample size may limit the generalizability of our findings to broader populations. To mitigate this limitation, we employed rigorous statistical analyses. Nevertheless, we recognize that these limitations may influence the interpretation of our results. Future long-term follow-up research with larger, controlled cohorts is warranted to validate and expand upon these findings, to address residual symptoms and psychological health challenges and assess the long-term durability of these benefits.

## 5. Conclusions

Our study demonstrates that DAA therapy significantly improves HRQoL in Romanian patients with chronic HCV infection, independent of gender or residence. While factors such as fibrosis degree and prior PegIFN treatment did not significantly influence HRQoL changes, specific comorbidities, notably chronic kidney disease and anemia, were identified as factors that moderated improvements in particular HRQoL domains, as was the presence of anxiety. Despite these overall positive outcomes, the persistence of some side effects warrants continued attention to patient management. From an academic perspective, our study contributes to the understanding of the complex interplay between the new DAA therapeutic approach and patients’ HRQoL, and opens new directions for future studies investigating not only clinical benefits, but also patients’ psychosocial well-being. For clinical practice, our study underscores the importance of personalized care strategies that address issues such as gender-specific comorbidities, as well as the importance of consistent monitoring for mental health conditions, which are important contributors to HRQoL, by integrating mental health assessments into routine HCV patient care.

## Figures and Tables

**Table 1:** Distribution of the study sample according to gender, age, and residence.

	Age Group				
Gender	<60 Years n (%)	60–69 Years n (%)		70–79 Years n (%)	* *p*
Females	13 (18.84)	42 (60.87)		14 (20.29)	0.553
Males	5 (23.81)	10 (47.62)		6 (28.57)	
Total	18 (20.00)	52 (57.78)		20 (22.22)	
	**Residence**				
	**Rural n (%)**		**Urban n (%)**		
Females	38 (55.07)		31 (44.93)		0.549
Males	10 (47.62)		11 (52.38)		
Total	48 (53.33)		42 (46.67)		

* Chi-square test.

**Table 2:** Distribution of the study sample according to gender, fibrosis degree, and prior PegIFN treatment.

	Fibrosis Degree				
	F2 n (%)	F3 n (%)		F4 n (%)	* *p*
Females	2 (2.90)	29 (42.03)		38 (55.07)	0.314
Males	1 (4.76)	5 (23.81)		15 (71.43)	
Total	3 (3.33)	34 (37.78)		53 (58.89)	
	**PegIFN Treatment**				
	**Yes n (%)**		**No n (%)**		
Females	19 (27.54)		50 (72.46)		0.6078
Males	7 (33.33)		14 (66.67)		
Total	26 (28.89)		64 (71.11)		

* Chi-square test.

**Table 3:** Distribution of the study sample according to gender and DAA therapy side effects at EOT and SVR.

		Side Effects			
	Study Stage	Fatigue n (%)	Headache n (%)	Insomnia n (%)	Pruritus n (%)
Females	EOT	6 (8.70)	5 (7.25)	6 (7.25)	7 (10.14)
	SVR	6 (8.70)	0 (0.00)	0 (0.00)	1 (1.45)
* *p*		1.000	0.058	**0.028**	**0.034**
Males	EOT	2 (9.52)	1 (4.76)	0 (0.00)	4 (19.05)
	SVR	1 (4.76)	0 (0.00)	0 (0.00)	1 (4.76)
* *p*		0.616	1.000	1.000	0.343
Total	EOT	8 (8.89)	6 (6.67)	5 (5.56)	11 (12.22)
	SVR	7 (7.78)	0 (0.00)	0 (0.00)	2 (2.22)
* *p*		1.000	**0.029**	0.059	**0.018**

* Fisher’s exact test.

**Table 4:** Distribution of the study sample according to gender and presence of comorbidities.

	Comorbidity		
	Yes n (%)	No n (%)	* *p*
	**Obesity**		
Female	53 (76.81)	16 (23.19)	0.351
Male	14 (66.67)	7 (33.33)	
Total	67 (74.44)	23 (25.56)	
	**Anemia**		
Female	29 (42.03)	40 (57.97)	0.651
Male	10 (47.62)	11 (52.38)	
Total	39 (43.33)	51 (56.67)	
	**Chronic Kidney Disease**		
Female	63 (91.30)	6 (8.70)	**0.0000012**
Male	9 (42.86)	12 (57.14)	
Total	72 (80.00)	18 (20.00)	
	**High Blood Pressure**		
Female	44 (63.77)	25 (36.23)	0.877
Male	13 (61.90)	8 (38.10)	
Total	57 (63.33)	33 (36.67)	
	**Diabetes**		
Female	14 (20.29)	55 (79.71)	0.095
Male	1 (4.76)	20 (95.24)	
Total	15 (16.67)	75 (83.33)	

* Chi-square.

**Table 5:** Distribution of the study sample according to gender, perceived stress, anxiety, and depression.

		Level of Perceived Stress (PSS)			
Stage	Gender	Low n (%)	Moderate n (%)	High n (%)	* *p*
BSL	Female	1 (1.45)	53 (76.81)	15 (21.74)	0.633
	Male	0 (0.00)	18 (85.71)	3 (14.29)	
EOT	Female	8 (11.59)	54 (78.26)	7 (10.14)	0.109
	Male	2 (9.52)	13 (61.90)	6 (28.57)	
SVR	Female	17 (24.64)	48 (69.57)	4 (5.80)	0.092
	Male	10 (47.62)	11 (52.38)	0 (0.00)	
		**Presence of Anxiety (HADS-A)**			
		**Normal** **n (%)**	**Borderline** **n (%)**	**Anxiety** **n (%)**	
BSL	Female	13 (18.84)	24 (34.78)	32 (46.38)	0.338
	Male	7 (33.33)	7 (33.33)	7 (33.33)	
EOT	Female	34 (49.28)	29 (42.03)	6 (8.70)	0.750
	Male	12 (57.14)	8 (38.10)	1 (4.76)	
SVR	Female	55 (79.71)	12 (17.39)	2 (2.90)	0.317
	Male	14 (66.67)	5 (23.81)	2 (9.52)	
		**Presence of Depression (HADS-D)**			
		**Normal** **n (%)**	**Borderline** **n (%)**	**Depression** **n (%)**	
BSL	Female	41 (59.42)	16 (23.19)	12 (17.39)	0.098
	Male	7 (33.33)	7 (33.33)	7 (33.33)	
EOT	Female	64 (92.75)	3 (4.35)	2 (2.90)	0.731
	Male	20 (95.24)	1 (4.76)	0 (0.00)	
SVR	Female	63 (91.30)	6 (8.70)	0 (0.00)	0.187
	Male	18 (85.71)	2 (9.52)	1 (4.76)	

* Chi-square.

**Table 6:** The evolution of WHOQOL BREF scores during the study period.

	WHOQOL BREF Average Score			
**Females**				
**WHOQOL BREF Domain**	**BSL**	**EOT**	**SVR**	*p* *
Overall QoL and general health	6.32 ± 1.44	7.51 ± 1.21	8.01 ± 1.24	**<0.0001**
Physical	58.36 ± 17.51	64.28 ± 14.93	74.13 ± 15.22	**<0.0001**
Psychological	67.72 ± 13.47	74.39 ± 12.42	82.25 ± 14.54	**<0.0001**
Social	53.38 ± 19.96	63.52 ± 15.90	72.68 ± 14.25	**<0.0001**
Environment	66.07 ± 12.69	73.04 ± 12.31	79.64 ± 11.76	**<0.0001**
**Males**				
**WHOQOL BREF Domain**	**BSL**	**EOT**	**SVR**	*p* *
Overall QoL and general health	6.43 ± 1.16	7.38 ± 1.02	7.86 ± 1.31	**<0.0001**
Physical	55.24 ± 13.30	64.33 ± 11.48	68.95 ± 13.28	**<0.0001**
Psychological	70.00 ± 13.74	78.05 ± 9.92	80.00 ± 13.26	**<0.0001**
Social	52.67 ± 18.73	66.10 ± 12.07	72.62 ± 15.23	**<0.0001**
Environment	66.38 ± 8.15	76.00 ± 9.76	78.43 ± 9.92	**<0.0001**
**Total**				
**WHOQOL BREF Domain**	**BSL**	**EOT**	**SVR**	*p* *
Overall QoL and general health	6.34 ± 1.37	7.48 ± 1.16	7.98 ± 1.25	**<0.0001**
Physical	57.63 ± 16.51	64.29 ± 14.06	72.92 ± 14.80	**<0.0001**
Psychological	68.26 ± 13.41	75.24 ± 11.86	81.72 ± 14.13	**<0.0001**
Social	53.21 ± 19.47	64.12 ± 14.98	72.67 ± 14.31	**<0.0001**
Environment	66.14 ± 11.68	73.73 ± 11.72	79.36 ± 11.25	**<0.0001**

* Friedman’s test.

**Table 7:** The comparison of WHOQOL BREF scores differences between the study stages for gender and residence.

		Mann–Whitney *p*		
	WHOQOL BREF Domains	EOT vs. BSL	SVR vs. EOT	SVR vs. BSL
Gender	Overall QoL and general health	0.587	0.485	0.474
	Physical	0.464	0.248	0.796
	Psychological	0.833	0.105	0.436
	Social	0.455	0.382	0.762
	Environment	0.392	0.313	0.733
Residence	Overall QoL and general health	0.243	0.667	0.514
	Physical	0.165	0.735	0.097
	Psychological	0.250	0.702	0.580
	Social	0.583	0.370	1.000
	Environment	0.076	0.283	0.504

**Table 8:** The comparison of WHOQOL BREF scores differences between the study stages for clinical data.

		Mann–Whitney *p*		
	WHOQOL BREF Domains	EOT vs. BSL	SVR vs. EOT	SVR vs. BSL
Fibrosis degree	Overall QoL and general health	0.628	0.217	0.664
	Physical	0.536	0.439	0.881
	Psychological	1.000	0.903	0.827
	Social	0.722	0.586	0.732
	Environment	0.296	0.231	0.834
PegIFN treatment	Overall QoL and general health	0.917	0.981	0.414
	Physical	0.266	0.699	0.982
	Psychological	0.380	0.924	0.881
	Social	0.188	0.335	0.103
	Environment	0.339	0.072	0.892
Side effects	Overall QoL and general health	0.928	0.531	0.682
	Physical	0.531	0.827	0.327
	Psychological	0.099	0.301	0.710
	Social	0.415	**0.048**	0.432
	Environment	**0.016**	**0.015**	0.457

**Table 9:** The comparison of WHOQOL BREF score differences between the study stages for comorbidities.

		Mann–Whitney *p*		
	WHOQOL BREF Domains	EOT vs. BSL	SVR vs. EOT	SVR vs. BSL
Obesity	Overall QoL and general health	0.323	0.240	0.979
	Physical	0.171	0.079	0.342
	Psychological	0.109	0.079	0.903
	Social	0.436	0.055	0.685
	Environment	**0.047**	**0.044**	0.664
Anaemia	Overall QoL and general health	0.513	**0.040**	0.313
	Physical	0.772	**0.017**	0.056
	Psychological	0.967	**0.018**	0.181
	Social	0.954	0.051	0.182
	Environment	0.935	**0.003**	**0.037**
Chronic Kidney Disease	Overall QoL and general health	0.139	0.065	0.802
	Physical	0.261	0.931	0.460
	Psychological	0.313	0.254	0.972
	Social	**0.003**	**0.038**	0.134
	Environment	0.103	0.666	0.390
High Blood Pressure	Overall QoL and general health	0.322	0.320	0.662
	Physical	0.075	0.082	0.857
	Psychological	0.139	0.186	0.625
	Social	0.366	0.317	0.827
	Environment	0.447	0.151	0.578
Diabetes	Overall QoL and general health	0.189	0.535	0.410
	Physical	0.680	0.495	0.539
	Psychological	0.332	0.335	0.935
	Social	**0.045**	0.053	0.624
	Environment	0.302	0.166	0.362

**Table 10:** The evolution of WHOQOL BREF scores during the study period based on psychological status.

		Mann–Whitney *p*		
	WHOQOL BREF Domains	EOT vs. BSL	SVR vs. EOT	SVR vs. INIT
Perceived stress	Overall QoL and general health	0.207	0.068	0.154
	Physical	0.445	0.280	**0.032**
	Psychological	0.613	0.323	0.268
	Social	0.185	0.223	0.199
	Environment	0.647	0.932	0.665
Anxiety	Overall QoL and general health	0.210	0.701	0.996
	Physical	0.077	0.539	0.772
	Psychological	**0.002**	0.103	0.379
	Social	0.112	0.909	0.559
	Environment	**0.021**	0.605	0.429
Depression	Overall QoL and general health	0.405	0.853	0.355
	Physical	0.690	0.890	0.362
	Psychological	0.673	0.246	0.761
	Social	0.646	0.788	0.323
	Environment	0.697	0.296	0.898

## Data Availability

The data used in this study could be available upon a reasonable request and after the approval of the local IRB.
